# Supplementary material for: Augmenting the technology acceptance model with trust model for the initial adoption of a blockchain-based system
Source: PeerJ Comput Sci. 2021 May 21;7:e502. doi: 10.7717/peerj-cs.502 (PMC8157082; doi:10.7717/peerj-cs.502)
Supplement: Supplemental Information 1 [file peerj-cs-07-502-s001.pdf]

Survey Questionnaire for Pretest

* 1. Please provide a random ID. You may generate a random ID on this page.

Keep a note of this ID as you need to put the same ID in the next survey.

* 2. Please indicate your Gender

- Female
- Male
- Other

* 3. Please indicate your Age

- 18 to 24
- 25 to 34
- 35 to 44
- 45 to 54
- 55 to 64
- 65 to 74
- 75 or older

* 4. Please indicate your highest education level

- Graduated from high school
- Bachelors
- Masters
- PhD
- Other (please specify)

5. Which of the following best describes your current occupation/field of study?

- Business and Financial Operations Occupations
- Computer Science
- Engineering
- Life, Physical, and Social Science
- Arts, Design, Entertainment, Sports, and Media
- Education
- Other Sciences
- Other (please specify)

* 6. What continent are you from?

- Africa
- Asia
- Europe
- Oceania
- North America
- South America

* 7. I am familiar with research social networks (e.g. ResearchGate, LinkedIn, ORCID).

strongly disagree	moderately disagree	slightly disagree	neither	slightly agree	moderately agree	strongly agree

* 8. I am familiar with online shopping sites (e.g. Disney Store, Walmart).

strongly disagree	moderately disagree	slightly disagree	neither	slightly agree	moderately agree	strongly agree

* 9. I am familiar with blockchain and smart contracts.

strongly disagree	moderately disagree	slightly disagree	neither	slightly agree	moderately agree	strongly agree

We want to introduce you to the advantages of blockchain and smart contract technologies, how they

can improve the applications you know and are familiar with, namely online shopping. Here is a video that explains blockchain and smart contract technologies on an abstract level (please watch the video with HD quality in full screen).

Summary of the video: **Blockchain is a secure chain of digital ledger existing on multiple computers simultaneously such that no records on the ledger can be erased or edited. It is an unalterable distributed ledger.**
And, the smart contract is a self-executing contract that stores rules to verify and execute the agreed terms for executing some actions (e.g. accessing the records). Thus, these rules can define who gets access to the stored records, under what conditions, for example, for what declared purpose, in exchange of what (payment or virtual credit) and they allow every access to the data to be recorded. With the smart contracts and the blockchain, users can enjoy the increased transparency and protection of data from falling into the wrong hands.

Please answer all the pretest survey questions before using the system.

* 10. I believe that the information I provide to blockchain-based systems will be handled by appropriate processes.

strongly disagree	moderately disagree	slightly disagree	neither	slightly agree	moderately agree	strongly agree
○	○	○	○	○	○	○

* 11. I believe that the information I provide to the blockchain-based systems will be stored securely.

strongly disagree	moderately disagree	slightly disagree	neither	slightly agree	moderately agree	strongly agree
○	○	○	○	○	○	○

* 12. I believe that only legitimate organizations can view the information I provide to blockchain-based systems.

strongly disagree	moderately disagree	slightly disagree	neither	slightly agree	moderately agree	strongly agree
○	○	○	○	○	○	○

* 13. I believe that the blockchain-based system is trustworthy.

strongly disagree	moderately disagree	slightly disagree	neither	slightly agree	moderately agree	strongly agree
○	○	○	○	○	○	○

* 14. I am confident in the blockchain-based system.

strongly disagree	moderately disagree	slightly disagree	neither	slightly agree	moderately agree	strongly agree
◯	◯	◯	◯	◯	◯	◯

* 15. The blockchain-based system protects my privacy.

strongly disagree	moderately disagree	slightly disagree	neither	slightly agree	moderately agree	strongly agree
◯	◯	◯	◯	◯	◯	◯

* 16. The blockchain-based system secures my information.

strongly disagree	moderately disagree	slightly disagree	neither	slightly agree	moderately agree	strongly agree
◯	◯	◯	◯	◯	◯	◯

* 17. The blockchain-based system has integrity.

strongly disagree	moderately disagree	slightly disagree	neither	slightly agree	moderately agree	strongly agree
◯	◯	◯	◯	◯	◯	◯

* 18. The blockchain-based system is dependable.

strongly disagree	moderately disagree	slightly disagree	neither	slightly agree	moderately agree	strongly agree
◯	◯	◯	◯	◯	◯	◯

* 19. The blockchain-based system can be relied on to keep its promises.

strongly disagree	moderately disagree	slightly disagree	neither	slightly agree	moderately agree	strongly agree
◯	◯	◯	◯	◯	◯	◯

* 20. I can trust a blockchain-based system.

strongly disagree	moderately disagree	slightly disagree	neither	slightly agree	moderately agree	strongly agree
◯	◯	◯	◯	◯	◯	◯

* 21. I am familiar with a blockchain-based system.

strongly disagree	moderately disagree	slightly disagree	neither	slightly agree	moderately agree	strongly agree
◯	◯	◯	◯	◯	◯	◯

* 22. I am aware of which organizations collect information I provide during the use of a blockchain-based system.

strongly disagree	moderately disagree	slightly disagree	neither	slightly agree	moderately agree	strongly agree
○	○	○	○	○	○	○

* 23. I am aware of the exact nature of the information that will be collected during the use of a blockchain-based system.

strongly disagree	moderately disagree	slightly disagree	neither	slightly agree	moderately agree	strongly agree
○	○	○	○	○	○	○

* 24. I believe that the information I put on a blockchain-based system can not be misused.

strongly disagree	moderately disagree	slightly disagree	neither	slightly agree	moderately agree	strongly agree
○	○	○	○	○	○	○

* 25. I believe that the blockchain accounts I use can not be intercepted by someone else.

strongly disagree	moderately disagree	slightly disagree	neither	slightly agree	moderately agree	strongly agree
○	○	○	○	○	○	○

This page measures your general behavior towards using online services.

* 26. I only register for web services that have a privacy policy.

strongly disagree	moderately disagree	slightly disagree	neither	slightly agree	moderately agree	strongly agree
○	○	○	○	○	○	○

* 27. I read the privacy policy of a web service before I register.

strongly disagree	moderately disagree	slightly disagree	neither	slightly agree	moderately agree	strongly agree
○	○	○	○	○	○	○

* 28. I look for a privacy certification of a web service before I register.

strongly disagree	moderately disagree	slightly disagree	neither	slightly agree	moderately agree	strongly agree
○	○	○	○	○	○	○

* 29. I read license agreements fully before I agree to them.

strongly disagree	moderately disagree	slightly disagree	neither	slightly agree	moderately agree	strongly agree
○	○	○	○	○	○	○

This page measures your general behavior towards using online services.

* 30. I regularly remove browser cookies.

strongly disagree	moderately disagree	slightly disagree	neither	slightly agree	moderately agree	strongly agree
○	○	○	○	○	○	○

* 31. I regularly use a pop-up window blocker.

strongly disagree	moderately disagree	slightly disagree	neither	slightly agree	moderately agree	strongly agree
○	○	○	○	○	○	○

* 32. I regularly check the computer for spyware.

strongly disagree	moderately disagree	slightly disagree	neither	slightly agree	moderately agree	strongly agree
○	○	○	○	○	○	○

* 33. I regularly clear my browser history.

strongly disagree	moderately disagree	slightly disagree	neither	slightly agree	moderately agree	strongly agree
○	○	○	○	○	○	○

* 34. I believe that using the blockchain-based system would be beneficial for me.

strongly disagree	moderately disagree	slightly disagree	neither	slightly agree	moderately agree	strongly agree
○	○	○	○	○	○	○

* 35. In my opinion, it would be desirable for me to use the blockchain-based system.

strongly disagree	moderately disagree	slightly disagree	neither	slightly agree	moderately agree	strongly agree
○	○	○	○	○	○	○

* 36. It would be good for me to use the blockchain-based system.

strongly disagree	moderately disagree	slightly disagree	neither	slightly agree	moderately agree	strongly agree
○	○	○	○	○	○	○

* 37. Do you have any other comments, questions, or concerns?
