# Supplementary material for: Augmenting the technology acceptance model with trust model for the initial adoption of a blockchain-based system
Source: PeerJ Comput Sci. 2021 May 21;7:e502. doi: 10.7717/peerj-cs.502 (PMC8157082; doi:10.7717/peerj-cs.502)
Supplement: Supplemental Information 3 [file peerj-cs-07-502-s003.pdf]

Survey Questionnaire for Data Sharing System (DSS)

* 1. Please provide **the ID** that you generated for the pretest survey

* 2. I am familiar with blockchain and smart contracts.

strongly disagree	moderately disagree	slightly disagree	neither	slightly agree	moderately agree	strongly agree
○	○	○	○	○	○	○

After completing the pretest survey and testing the second part of the system, you may now complete this questionnaire. If you want to see the video on how to use the second part of the system, please click this video link (please watch it with HD quality in full-screen mode).

Summary of the video: The proposed system enables enterprises to leverage the opportunity to receive features provided by the blockchain and smart contract technologies. This is the second part of the system and it allows companies such as Online Shopping-Cart Enterprise to share user data among other companies in their consortium network via blockchain and incentivize every customer with micro-payment for the use of their data. They can also obtain the provenance of every transaction.

Please answer the following questions to evaluate the second part of the proposed framework which is an example use case of the user-controlled privacy-preserving user data sharing framework based on blockchains and smart contracts.

* 3. Learning to operate this system is easy.

strongly disagree	moderately disagree	slightly disagree	neither	slightly agree	moderately agree	strongly agree
○	○	○	○	○	○	○

* 4. I find it easy to get this system to do what I want it to do.

strongly disagree	moderately disagree	slightly disagree	neither	slightly agree	moderately agree	strongly agree
○	○	○	○	○	○	○

* 5. My interaction with this system is clear and understandable.

strongly disagree	moderately disagree	slightly disagree	neither	slightly agree	moderately agree	strongly agree
○	○	○	○	○	○	○

* 6. I find this system to be flexible to interact with.

strongly disagree	moderately disagree	slightly disagree	neither	slightly agree	moderately agree	strongly agree
○	○	○	○	○	○	○

* 7. I feel it is easy to become skillful at using this system.

strongly disagree	moderately disagree	slightly disagree	neither	slightly agree	moderately agree	strongly agree
○	○	○	○	○	○	○

* 8. I find this system easy to use.

strongly disagree	moderately disagree	slightly disagree	neither	slightly agree	moderately agree	strongly agree
○	○	○	○	○	○	○

* 9. Using this system would enable me to share data more quickly in the consortium network.

strongly disagree	moderately disagree	slightly disagree	neither	slightly agree	moderately agree	strongly agree
○	○	○	○	○	○	○

* 10. Using this system would improve performance with data sharing.

strongly disagree	moderately disagree	slightly disagree	neither	slightly agree	moderately agree	strongly agree
○	○	○	○	○	○	○

* 11. Using this system would increase productivity with data sharing.

strongly disagree	moderately disagree	slightly disagree	neither	slightly agree	moderately agree	strongly agree
○	○	○	○	○	○	○

* 12. Using this system would increase effectiveness with data sharing.

strongly disagree	moderately disagree	slightly disagree	neither	slightly agree	moderately agree	strongly agree
◯	◯	◯	◯	◯	◯	◯

* 13. Using this system would make it easier to share and access data by incentivizing the data owners.

strongly disagree	moderately disagree	slightly disagree	neither	slightly agree	moderately agree	strongly agree
◯	◯	◯	◯	◯	◯	◯

* 14. I find this system useful for sharing and accessing user data by incentivizing the data owners.

strongly disagree	moderately disagree	slightly disagree	neither	slightly agree	moderately agree	strongly agree
◯	◯	◯	◯	◯	◯	◯

* 15. I am satisfied with the feature of sharing user data in the consortium blockchain network.

strongly disagree	moderately disagree	slightly disagree	neither	slightly agree	moderately agree	strongly agree
◯	◯	◯	◯	◯	◯	◯

* 16. I am satisfied with the methods of accessing user data by providing incentives to the data owners.

strongly disagree	moderately disagree	slightly disagree	neither	slightly agree	moderately agree	strongly agree
◯	◯	◯	◯	◯	◯	◯

* 17. I am satisfied with how the system grants access to user data after fulfilling the terms of smart contracts.

strongly disagree	moderately disagree	slightly disagree	neither	slightly agree	moderately agree	strongly agree
◯	◯	◯	◯	◯	◯	◯

* 18. I am satisfied with how the system creates a proof of the existence of transactions via smart contracts.

strongly disagree	moderately disagree	slightly disagree	neither	slightly agree	moderately agree	strongly agree
◯	◯	◯	◯	◯	◯	◯

* 19. I would like to use this system to share user data.

strongly disagree	moderately disagree	slightly disagree	neither	slightly agree	moderately agree	strongly agree
◯	◯	◯	◯	◯	◯	◯

* 20. I would like to follow smart contracts for accessing user data.

strongly disagree	moderately disagree	slightly disagree	neither	slightly agree	moderately agree	strongly agree
◯	◯	◯	◯	◯	◯	◯

* 21. I would enjoy using this system when I need to use it.

strongly disagree	moderately disagree	slightly disagree	neither	slightly agree	moderately agree	strongly agree
◯	◯	◯	◯	◯	◯	◯

* 22. It is worthwhile to use this system to share user data and provide incentives to the data owners for accessing their data.

strongly disagree	moderately disagree	slightly disagree	neither	slightly agree	moderately agree	strongly agree
◯	◯	◯	◯	◯	◯	◯

* 23. I will use this system to share user data and provide incentives to the data owners for accessing their data.

strongly disagree	moderately disagree	slightly disagree	neither	slightly agree	moderately agree	strongly agree
◯	◯	◯	◯	◯	◯	◯

* 24. I intend to use this system to share user data and provide incentives to the data owners for accessing their data.

strongly disagree	moderately disagree	slightly disagree	neither	slightly agree	moderately agree	strongly agree
◯	◯	◯	◯	◯	◯	◯

* 25. I believe appropriate processes will handle the information I provide with blockchain.

strongly disagree	moderately disagree	slightly disagree	neither	slightly agree	moderately agree	strongly agree
◯	◯	◯	◯	◯	◯	◯

* 26. I believe that the information I provide will be stored securely.

strongly disagree	moderately disagree	slightly disagree	neither	slightly agree	moderately agree	strongly agree
◯	◯	◯	◯	◯	◯	◯

* 27. I believe that only legitimate organizations can view the information I provide to this system.

strongly disagree	moderately disagree	slightly disagree	neither	slightly agree	moderately agree	strongly agree
◯	◯	◯	◯	◯	◯	◯

* 28. I believe that this blockchain-based system is trustworthy.

strongly disagree	moderately disagree	slightly disagree	neither	slightly agree	moderately agree	strongly agree
◯	◯	◯	◯	◯	◯	◯

* 29. This system can be relied on to keep its promises.

strongly disagree	moderately disagree	slightly disagree	neither	slightly agree	moderately agree	strongly agree
◯	◯	◯	◯	◯	◯	◯

* 30. This system is dependable.

strongly disagree	moderately disagree	slightly disagree	neither	slightly agree	moderately agree	strongly agree
◯	◯	◯	◯	◯	◯	◯

* 31. This system has integrity.

strongly disagree	moderately disagree	slightly disagree	neither	slightly agree	moderately agree	strongly agree
◯	◯	◯	◯	◯	◯	◯

* 32. This system protects my privacy.

strongly disagree	moderately disagree	slightly disagree	neither	slightly agree	moderately agree	strongly agree
○	○	○	○	○	○	○

* 33. This system secures my information.

strongly disagree	moderately disagree	slightly disagree	neither	slightly agree	moderately agree	strongly agree
○	○	○	○	○	○	○

* 34. I am familiar with this system.

strongly disagree	moderately disagree	slightly disagree	neither	slightly agree	moderately agree	strongly agree
○	○	○	○	○	○	○

* 35. I am confident in this system.

strongly disagree	moderately disagree	slightly disagree	neither	slightly agree	moderately agree	strongly agree
○	○	○	○	○	○	○

* 36. I can trust this system.

strongly disagree	moderately disagree	slightly disagree	neither	slightly agree	moderately agree	strongly agree
○	○	○	○	○	○	○

* 37. I am aware of which organizations access the user data during the use of this blockchain-based system.

strongly disagree	moderately disagree	slightly disagree	neither	slightly agree	moderately agree	strongly agree
○	○	○	○	○	○	○

* 38. I am aware of the exact nature of the information that will be collected during the use of this system.

strongly disagree	moderately disagree	slightly disagree	neither	slightly agree	moderately agree	strongly agree
○	○	○	○	○	○	○

* 39. I believe that the information I put on this system can not be misused.

strongly disagree	moderately disagree	slightly disagree	neither	slightly agree	moderately agree	strongly agree
○	○	○	○	○	○	○

* 40. I believe that the blockchain accounts that I use on this system can not be intercepted by someone else.

strongly disagree	moderately disagree	slightly disagree	neither	slightly agree	moderately agree	strongly agree
○	○	○	○	○	○	○

* 41. I believe that using the blockchain-based system would be beneficial for me.

strongly disagree	moderately disagree	slightly disagree	neither	slightly agree	moderately agree	strongly agree
○	○	○	○	○	○	○

* 42. In my opinion, it would be desirable for me to use the blockchain-based system.

strongly disagree	moderately disagree	slightly disagree	neither	slightly agree	moderately agree	strongly agree
○	○	○	○	○	○	○

* 43. It would be good for me to use the blockchain-based system.

strongly disagree	moderately disagree	slightly disagree	neither	slightly agree	moderately agree	strongly agree
○	○	○	○	○	○	○

* 44. Do you have any other comments, questions, or concerns?
