# Supplementary material for: Augmenting the technology acceptance model with trust model for the initial adoption of a blockchain-based system
Source: PeerJ Comput Sci. 2021 May 21;7:e502. doi: 10.7717/peerj-cs.502 (PMC8157082; doi:10.7717/peerj-cs.502)
Supplement: Supplemental Information 4 [file peerj-cs-07-502-s004.pdf]

Indicators	No.	Missing	Mean	Median	Min	Max	Standard Deviation	Excess Kurtosis	Skewness
PS1	1	0	5.88	6	3	7	0.986	0.708	-0.96
PS2	2	0	5.74	6	2	7	1.22	0.225	-0.904
PS3	3	0	5.19	6	1	7	1.513	-0.074	-0.807
T1	4	0	5.99	6	4	7	0.83	-0.505	-0.408
T2	5	0	5.74	6	4	7	0.945	-0.75	-0.34
T3	6	0	5.45	6	1	7	1.243	1.375	-0.952
T4	7	0	5.8	6	1	7	1.078	3.779	-1.301
T5	8	0	6	6	4	7	0.915	-0.612	-0.54
T6	9	0	5.64	6	3	7	1.06	-0.762	-0.468
T7	10	0	5.76	6	1	7	1.076	3.75	-1.357
T8	11	0	5.77	6	4	7	0.966	-0.724	-0.436
T9	12	0	5.2	5	1	7	1.345	0.405	-0.754
AP1	13	0	4.81	5	2	7	1.458	-1.006	-0.09
AP2	14	0	4.82	5	1	7	1.501	-0.3	-0.55
AP3	15	0	5.05	6	1	7	1.593	-0.454	-0.705
AP4	16	0	5.3	6	2	7	1.495	-0.807	-0.576
BP-GC1	17	0	5.32	6	1	7	1.603	0.588	-1.05
BP-GC2	18	0	4.22	4	1	7	1.773	-0.927	-0.306
BP-GC3	19	0	4.49	5	1	7	1.854	-0.909	-0.238
BP-GC4	20	0	3.5	3	1	7	1.803	-0.979	0.29
BP-TP1	21	0	5.01	5	1	7	1.681	0.094	-0.981
BP-TP2	22	0	5.93	6	2	7	1.082	1.655	-1.104
BP-TP3	23	0	5.3	6	1	7	1.625	0.062	-0.939
BP-TP4	24	0	5.14	6	1	7	1.765	0.129	-1.052
ATS1	25	0	5.68	6	2	7	1.092	0.292	-0.717
ATS2	26	0	5.57	6	2	7	1.128	0.036	-0.633
ATS3	27	0	5.61	6	3	7	1.076	-0.897	-0.353
