# Supplementary material for: Augmenting the technology acceptance model with trust model for the initial adoption of a blockchain-based system
Source: PeerJ Comput Sci. 2021 May 21;7:e502. doi: 10.7717/peerj-cs.502 (PMC8157082; doi:10.7717/peerj-cs.502)
Supplement: Supplemental Information 5 [file peerj-cs-07-502-s005.pdf]

	No.	Missing	Mean	Median	Min	Max	Standard Deviation	Excess Kurtosis	Skewness
PEOU1	1	0	6.254	6	5	7	0.689	-0.855	-0.389
PEOU2	2	0	6.063	6	5	7	0.774	-1.334	-0.112
PEOU3	3	0	6.19	6	3	7	0.94	4.156	-1.804
PEOU4	4	0	6.063	6	1	7	0.906	14.264	-2.75
PEOU5	5	0	6.365	6	4	7	0.741	1.525	-1.197
PEOU6	6	0	6.365	6	5	7	0.697	-0.726	-0.652
PU1	7	0	6.032	6	3	7	0.975	0.632	-1.011
PU2	8	0	6	6	3	7	0.926	0.644	-0.861
PU3	9	0	5.921	6	1	7	1.088	5.842	-1.883
PU4	10	0	6.365	6	4	7	0.674	1.057	-0.925
PU5	11	0	6.381	7	5	7	0.7	-0.692	-0.7
PU6	12	0	6.413	7	5	7	0.705	-0.6	-0.797
QOS1	13	0	6.19	6	4	7	0.794	0.092	-0.752
QOS2	14	0	6.238	6	4	7	0.728	0.052	-0.662
QOS3	15	0	6.381	6	4	7	0.677	1.088	-0.969
QOS4	16	0	6.397	7	4	7	0.746	0.439	-1.045
Enj1	17	0	6.079	6	3	7	0.803	2.421	-1.091
Enj2	18	0	6.302	6	4	7	0.769	0.805	-1.017
Enj3	19	0	6.19	6	4	7	0.814	-0.115	-0.733
ITU1	20	0	6.063	6	4	7	0.833	-0.131	-0.628
ITU2	21	0	6.063	6	4	7	0.889	-0.273	-0.684
ITU3	22	0	6.063	6	3	7	0.871	1.847	-1.161
PS1	23	0	5.73	6	3	7	0.84	0.596	-0.434
PS2	24	0	5.841	6	4	7	0.963	-0.75	-0.436
PS3	25	0	5.794	6	3	7	1.041	0.035	-0.694
T1	26	0	6.111	6	5	7	0.645	-0.582	-0.111
T2	27	0	6.016	6	3	7	0.917	1.133	-1.044
T3	28	0	5.81	6	3	7	0.99	-0.159	-0.609
T4	29	0	6.159	6	4	7	0.801	-0.077	-0.68
T5	30	0	5.937	6	3	7	0.871	0.894	-0.761
T6	31	0	5.905	6	4	7	0.849	-0.336	-0.451
T7	32	0	5.841	6	3	7	0.912	0.46	-0.703
T8	33	0	5.889	6	3	7	0.875	0.653	-0.652
T9	34	0	5.921	6	4	7	0.841	-0.216	-0.501
AP1	35	0	5.762	6	4	7	0.904	-0.82	-0.163
AP2	36	0	5.667	6	3	7	1.054	0.167	-0.704
AP3	37	0	5.397	5	3	7	1.134	-0.793	-0.239
AP4	38	0	5.492	6	3	7	1.344	-0.589	-0.767
ATS1	39	0	6.048	6	4	7	0.765	0.028	-0.519
ATS2	40	0	5.952	6	4	7	0.785	-0.406	-0.317
ATS3	41	0	6	6	4	7	0.797	-0.419	-0.386
