# Supplementary material for: Augmenting the technology acceptance model with trust model for the initial adoption of a blockchain-based system
Source: PeerJ Comput Sci. 2021 May 21;7:e502. doi: 10.7717/peerj-cs.502 (PMC8157082; doi:10.7717/peerj-cs.502)
Supplement: Supplemental Information 6 [file peerj-cs-07-502-s006.pdf]

	No.	Missing	Mean	Median	Min	Max	Standard Deviation	Excess Kurtosis	Skewness
PEOU1	1	0	5.96	6	3	7	0.916	0.63	-0.724
PEOU2	2	0	5.72	6	3	7	1.217	0.181	-0.945
PEOU3	3	0	5.86	6	4	7	0.895	-0.849	-0.232
PEOU4	4	0	5.48	6	2	7	1.22	0.019	-0.599
PEOU5	5	0	5.94	6	3	7	1.047	1.686	-1.278
PEOU6	6	0	5.72	6	2	7	1.201	1.163	-1.077
PU1	7	0	5.9	6	3	7	1.204	0.535	-1.148
PU2	8	0	5.62	6	1	7	1.384	1.078	-1.144
PU3	9	0	5.7	6	1	7	1.345	1.562	-1.204
PU4	10	0	5.88	6	4	7	0.993	-0.772	-0.51
PU5	11	0	6.28	6	4	7	0.776	1.143	-1.077
PU6	12	0	6.34	7	4	7	0.764	0.33	-0.959
QOS1	13	0	5.82	6	3	7	1.052	-0.263	-0.687
QOS2	14	0	6.16	6	4	7	0.784	-0.403	-0.555
QOS3	15	0	6.14	6	4	7	0.825	-0.776	-0.493
QOS4	16	0	6.42	7	4	7	0.666	1.98	-1.162
Enj1	17	0	5.9	6	3	7	0.985	0.356	-0.829
Enj2	18	0	6.1	6	4	7	0.831	-0.22	-0.626
Enj3	19	0	5.92	6	3	7	1.017	0.279	-0.894
ITU1	20	0	6.16	6	3	7	0.88	2.132	-1.234
ITU2	21	0	5.92	6	2	7	1.036	2.685	-1.281
ITU3	22	0	5.94	6	2	7	1.121	2.241	-1.372
PS1	23	0	5.94	6	3	7	0.925	0.751	-0.814
PS2	24	0	5.96	6	2	7	1.038	3.738	-1.575
PS3	25	0	5.9	6	3	7	1.1	0.878	-1.097
T1	26	0	6.3	6	4	7	0.755	0.271	-0.863
T2	27	0	6.22	6	4	7	0.782	-0.241	-0.678
T3	28	0	6.06	6	3	7	0.968	0.985	-1.08
T4	29	0	6.32	6	4	7	0.76	0.295	-0.91
T5	30	0	5.96	6	3	7	0.916	2.43	-1.208
T6	31	0	5.9	6	3	7	1.005	0.127	-0.768
T7	32	0	6.04	6	4	7	0.894	0.001	-0.775
T8	33	0	5.9	6	3	7	0.964	0.368	-0.759
T9	34	0	6.1	6	4	7	0.831	-0.22	-0.626
AP1	35	0	6.3	7	4	7	0.806	-0.206	-0.85
AP2	36	0	6.16	6	3	7	0.946	1.429	-1.211
AP3	37	0	5.32	6	1	7	1.378	0.58	-0.937
AP4	38	0	5.56	6	2	7	1.344	0.089	-0.82
ATS1	39	0	5.92	6	4	7	0.891	-0.713	-0.363
ATS2	40	0	5.76	6	3	7	1.05	-0.3	-0.67
ATS3	41	0	5.9	6	4	7	1.005	-0.603	-0.646
